# Haplotypes versus genotypes on pedigrees

**DOI:** 10.1186/1748-7188-6-10

**Published:** 2011-04-19

**Authors:** Bonnie B Kirkpatrick

**Affiliations:** 1 Electrical Engineering and Computer Sciences, University of California Berkeley, Berkeley, CA 94720-1776, USA; 2 International Computer Science Institute, 1947 Center St. Suite 600, Berkeley, CA 94704, USA

## Abstract

**Background:**

Genome sequencing will soon produce haplotype data for individuals. For pedigrees of related individuals, sequencing appears to be an attractive alternative to genotyping. However, methods for pedigree analysis with haplotype data have not yet been developed, and the computational complexity of such problems has been an open question. Furthermore, it is not clear in which scenarios haplotype data would provide better estimates than genotype data for quantities such as recombination rates.

**Results:**

To answer these questions, a reduction is given from genotype problem instances to haplotype problem instances, and it is shown that solving the haplotype problem yields the solution to the genotype problem, up to constant factors or coefficients. The pedigree analysis problems we will consider are the likelihood, maximum probability haplotype, and minimum recombination haplotype problems.

**Conclusions:**

Two algorithms are introduced: an exponential-time hidden Markov model (HMM) for haplotype data where some individuals are untyped, and a linear-time algorithm for pedigrees having haplotype data for all individuals. Recombination estimates from the general haplotype HMM algorithm are compared to recombination estimates produced by a genotype HMM. Having haplotype data on all individuals produces better estimates. However, having several untyped individuals can drastically reduce the utility of haplotype data.

## 

Pedigree analysis, both linkage and association studies, has a long history of important contributions to genetics, including disease-gene finding and some of the first genetic maps for humans. Recent contributions include fine-scale recombination maps in humans [1], regions linked to Schizophrenia that might be missed by genome-wide association studies [2], and insights into the relationship between cystic fibrosis and fertility [3]. Algorithms for pedigree problems are of great interest to the computer science community, in part because of connections to machine learning algorithms, optimization methods, and combinatorics [4-8].

Single-molecule sequencing is an attractive alternative to genotyping and would yield haplotypes for individuals in a pedigree [9]. Such technologies are being developed and may become commercial within five to ten years. Sequencing methods would apparently yield more information from the same set of sampled individuals, which is critical due to the limited availability of individuals for sampling in multi-generational pedigrees (i.e. individuals usually must be living at the time of sampling). There is substantial evidence that haplotypes can be more useful than genotypes for both population and family based studies when using methods such as association studies [10,11] and pedigree analysis [12,13]. While it is intuitive that haplotypes provide more information than genotypes, there are instances with family data in which there are few enough typed individuals that there is little practical difference between haplotype and genotype data. Additionally, in order to exploit the information contained in haplotype data, we need to understand the instances where diploid inheritance is computationally tractable given haplotype data.

Pedigree analysis with genotype data is well studied in terms of complexity [6,7] and algorithms [14-16]. Less is known about haplotype data on pedigrees. This paper shows that, given haplotype data on a pedigree, finding both minimum recombination and maximum probability haplotypes is as tractable as computing the same quantities for pedigrees with genotype data (i.e., these problems are NP- and #P-hard, respectively). To obtain a reduction that applies equally well to several types of pedigree calculations, we will consider a modular polynomial-time mapping from the genotype problem to the haplotype problem. The reduction preserves the solutions to the analyses, meaning that the solution to the haplotype problem is the solution to the genotype problem after adjusting by constant factors or coefficients.

Since the reduction uses a biologically unlikely recombination scenario, we will investigate the accuracy and information of realistic examples with haplotypes and genotype data on the same pedigree. Pedigree data was simulated having a known number of recombinations. The recombination distributions were computed at a particular locus of interest and compared to the ground-truth. Since both the haplotypes and genotypes of a specific person contain the same alleles, the differences between the haplotype and genotype recombination distributions were determined by the extra information in the haplotype data. As expected, the haplotype data reliably yields greater accuracy when all the pedigree individuals are typed. However, as fewer pedigree individuals are typed, there is less practical difference between the utility of haplotype versus genotype data. The number of untyped generations that separate typed individuals influences whether haplotype data are actually more accurate than genotype data. For instance with two half-siblings, having two untyped parents results in estimates from genotype data that are nearly as accurate as the estimates computed from haplotype data.

Finally, there is an important instance where haplotype data is more computationally tractable than genotype data. When all individuals in the pedigree are typed, although unlikely from a practical perspective of obtaining genetic samples, the computational problem decomposes into conditionally independent sub-problems, and has a linear-time algorithm. This can be contrasted with the known hardness of the genotype problem even when all individuals are genotyped. The existence of this linear-time algorithm for haplotype data could facilitate useful approaches that combine population genetic and pedigree methods. For instance, if the haplotypes of the founders are drawn from a coalescent and the pedigree individuals are all haplotyped, the probability of a combined model could easily be computed for certain coalescent models.

## Introduction to Pedigree Analysis

A *pedigree *is a directed acyclic graph where the set of nodes, *I*, are individuals, and directed edges indicate genetic inheritance between parent and child. A diploid pedigree (i.e. for humans) necessarily has either zero or two incoming edges for each person. The set, *F *, of individuals without incoming edges are referred to as pedigree *founders*. An individual, *i*, with two parents is a *non-founder*, and we will refer to their two parents as *m*(*i*) and *p*(*i*).

As is commonly done to accommodate inheritance of genetic information, we will extend this model to include a representation of the alleles of each individual and of the inheritance origin of each allele. More formally, we represent a single chromosome as an ordered sequence of variables, *x_j_*, where each variable takes on an *allele *value in {1, ..., *k_j_*}. Each variable represents a *polymorphic site*, *j*, in the genome, where there are *k_j _*possible sequence variants. Since diploid individuals have two copies of each chromosome, one copy inherited from each parent, we will use a superscript *m *and *p *to indicate the maternal and paternal chromosomes respectively. For a particular individual *i*, the information on both copies of a particular chromosome at site *j *is represented as  and .

Furthermore, we assume that inheritance in the pedigree proceeds with recombination and without mutation (i.e. Mendelian inheritance at each site). This imposes consistency rules on parents and children: the allele  must appear in the mother *m*(*i*)'s genome as either the grand-maternal or grand-paternal allele,  or , and similarly for the paternal allele and the father *p*(*i*)'s genome.

Let *x *be a vector containing all the haplotypes  for all individuals *i *∈ *I*, then we are interested in the probability

where the superscript *m *and *p *indicate maternal and paternal alleles, while the functions *m*(*i*) and *p*(*i*) indicate parents of *i*. The first product is over the independent founder individuals whose haplotypes are drawn from a uniform prior distribution, while the second product, over the non-founders, contains the probabilities for the children to inherit their haplotypes from their parents. The unobserved vector *x *is not immediately derived from observed haplotype data, since vector *x *contains haplotype alleles labeled with their parental origins for all the individuals. To compute this quantity, we need notation to represent the parental origins of each allele where differing origins for neighboring haplotype alleles will indicate recombination events.

For each non-founder, let us indicate the source of each maternal allele using the binary variable , where the value *m *indicates that  allele has grand-maternal origin and *p *indicates grand-paternal origin. Similarly, we define  for the origin of *i*'s paternal allele. For a particular site, these indicators for all the individuals, *s_j_*, is commonly referred to as the identity-by-descent (IBD) inheritance path. A recombination is observed at consecutive sites as a change in the binary value of a source vector, for instance,  and . To compute the inheritance portion of the equation for *P *[*x*], we will sum over the inheritance options ℙ[*x*] = ∑_*s *_ℙ[*x*|*s*] ℙ*s *where ℙ [*s*] = 1/2^2|*I*\*F*| ^We can observe two kinds of data for pedigree individuals whose genetic material is available. The first, and most common, is genotype data, a tuple of alleles  that must appear in the variables  and  for each site *j*. Since these alleles are unlabeled for origin, we do not know which allele was inherited from which parent. The second type of data is haplotypes, where we observe two sequences of alleles  and  and each sequence represents alleles that were inherited together from the same parent. However, we do not know which sequence is maternal and which is paternal. For either type of data define a function *C_i, j _*for locus *j *which indicates compatibility of the assigned haplotype alleles with the data and requires inheritance consistency between generations. Specifically, for genotype data *C_i, j _*= 1 if , , and . Under haplotype data, the *C_i, j _*= 1 when the first two equalities, above, hold and , which are the haplotype alleles at locus *j*.

Now, we write the equation for *P *[*x*] as a function of the per-site recombination probability *θ *≤ 0.5. For particular values of all the haplotype alleles  and , the haplotype probability conditional on the inheritance options and the observed data through *C_i, j _*is

where  and .

### Pedigree Problem Formulations

Given a pedigree and some observed genotype or haplotype data, there are three problem formulations that we might be interested in. The first is to compute the probability of some observed data, while the last two problems find values for the unobserved haplotypes of individuals in the pedigree.

#### Likelihood

Find the probability of the observed data by summing over all the possible unobserved haplotypes, i.e. ∑_*s *_∑_*s *_ℙ [*x*|*s*] ℙ [*s*].

#### Maximum Probability

Find the values of  and  that maximize the probability of the data, i.e. max_*x *_∑_*s *_ℙ [*x*|*s*] ℙ [*s*].

#### Minimum Recombination

Find the values of  and  that minimize the number of required recombinations, i.e. .

The likelihood is commonly used for estimating site-specific recombination rates, relationship testing, computing p-values for association tests, and performing linkage analysis. Haplotype and/or IBD inferences, obtained by maximizing the probability or minimizing the recombinations, are useful for non-parametric association tests, tests on haplotypes, and tests where there is disease information for unobserved genomes.

## Hardness Results

With genotype data, the likelihood and minimum recombination problems are NP-hard, while the maximum probability problem is #P-hard. Piccolboni and Gusfield [6] proved the hardness of the likelihood and maximum probability computations by relying on a single locus sub-pedigree with half-siblings. Although their paper discussed a more elaborate setting involving a phenotype, their proof, however, applies to this setting. Li and Jiang proved the minimum recombination problem to be hard by using a two-locus sub-pedigree with half-siblings [7]. In all these proofs, half-siblings were pivotal to establishing reductions from well known NP and #P problems.

In this paper, we introduce a simple and powerful reduction that converts any genotype problem on a pedigree of *n *individuals into a haplotype problem on a pedigree of at most 6*n *individuals. This reduction is simple, because it merely introduces four full-siblings and an extra parent for each genotyped individual. We do not need complicated structures involving inbreeding or half-siblings. The reduction works equally well for all three problem formulations.

### Mapping

Given a pedigree with genotype data, for any of the three pedigree problems, we define a polynomial mapping to a corresponding haplotype problem with exactly 5|*G*| individuals haplotyped. First we create the pedigree graph for the new haplotype instance, and later we construct the required haplotype observations from the genotype data.

**Figure 1:**
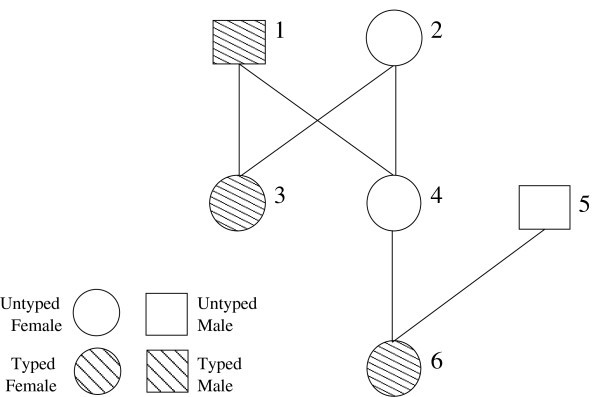
**Genotype and Haplotype Pedigrees**. Genotyped individuals are shaded, and all the individuals are labeled. Individuals 1, 2, and 5 are the founders, and individual 6 is the grandchild of 1 and 2.

**Figure 2:**
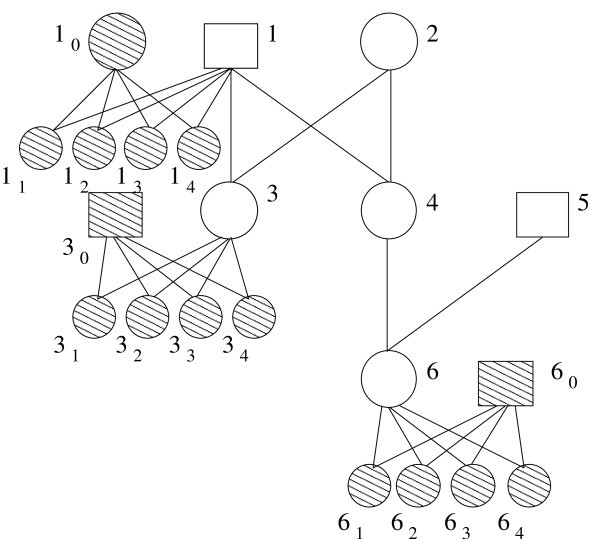
**Haplotype Pedigrees**. Haplotyped individuals are shaded, and individuals have the same labels. For each of the genotyped individuals, *i*, from the previous figure, the mapping adds a nuclear family containing five new individuals labeled *i*_0_, *i*_1_, *i*_2_, *i*_3_, *i*_4_.

Let *G *⊂ *I *represent the set of genotyped individuals in a pedigree having individuals *I *and edges *E*. We will create a haplotype instance of the problem, with individuals *H *∪ *I *and edges *R *∪ *E*. To obtain the set *H*, we add five individuals, *i*_0_, *i*_1_, *i*_2_, *i*_3_, *i*_4_, to *H *for every individual *i *∈ *G*. The set of new relationship edges, *R*, will connect individuals in sets *H *and *G*. Specifically, the edges stipulate that *i *and *i*_0 _are the parents of full-siblings *i*_1_, *i*_2_, *i*_3_, and *i*_4 _by including the edges: *i*_0 _→ *i*_1_, *i*_0 _→ *i*_2_, *i*_0 _→ *i*_3_, *i*_0 _→ *i*_4_, *i *→ *i*_1_, *i *→ *i*_2_, *i *→ *i*_3_, and *i *→ *i*_4_. We will refer to these five individuals, *i*_0_, *i*_1_, *i*_2_, *i*_3_, and *i*_4_, and their relationships with *i *as the *proxy family *for individual *i*. For example, the 6-individual genotype pedigree in Figure 1 becomes a 21-individual genotype pedigree in Figure 2. This produces a pedigree graph with exactly 5|*G*| + |*I *| individuals and 8|*G*| + |*E*| edges.

To obtain the new haplotype data from the genotype data, we type only individuals in *H *such that the corresponding genotyped individual in *G *is required, by the rules of inheritance, to have the observed genotypes. Without loss of generality, assume that the genotype alleles are sorted such that . Now we can easily constrain the parental genotype for individual *i *∈ *G *by giving the spouse, *i*_0_, homozygous haplotypes of all ones while giving child *i*_1 _the haplotypes , child *i*_2 _haplotypes . This guarantees the correct genotype, but does not ensure that the haplotypes of that genotype have the same probability or number of recombinations.

Since there is an arbitrary assorting of genotype alleles at neighboring loci into the parent haplotypes  and , we will use the remaining two children to represent possible re-assortments of the genotyped parent's *T_i _*heterozygous loci, indexed by *t_j _*where 1 ≤ *j *≤ *T_i_*. In addition to the haplotype , child *i*_3_, will have haplotype consisting of . while child *i*_4 _has the genotyped parent's complementary alleles . This results in child *i*_3 _and *i*_4 _alternating in having the smaller allele at every other heterozygous locus.

This reduction preserves the solutions to the three problems up to constant factors or constant coefficients. Specifically, the solution to the haplotype version of the problem is the solution to the genotype version with the values of the functions being related by constant factors or coefficients, depending on whether the function is a recombination count or a probability.

**Lemma 1**. *Let r_g _be the minimum number of recombinations in the genotype problem instance. The mapping yields a haplotype problem instance having*(1)

*for the minimum number of recombinations, where T_i _is the number of heterozygous sites in genotype i*.

To prove this result, we exploit the alternating pattern of alleles assigned to the four children. This pattern forces there to be two recombinations, among the four children, between consecutive heterozygous loci.

*Proof*. Consider the haplotype instance of the problem. Recall that set *G *is defined as the individuals who are genotyped in the genotype problem instance, and, by construction, they are not haplotyped in the haplotype problem instance. For each *i *∈ *G *the rules of inheritance applied to *i*'s proxy family dictate that the set of alleles at each position are given by  and . Therefore, the proxy family dictates the genotype of *i*.

Since the haplotypes for all the typed individuals are completely given, we only need to consider the assortment of the alleles from  and  into the maternal and paternal alleles of individual *i*. Clearly this assortment determines the number of recombinations that the proxy family contributes to Eqn. (1). However, we will use induction along the genome to show that every possible phasing of the parental genotype induces the same minimum number of recombinations among the four children, namely 2(*T_i _*- 1).

Now we define an arbitrary assortment of the genotype alleles into two haplotypes for person *i*. We can think of this parental genotype for *l *loci as a string *s *∈ {*H*, *T *}*^l^*, where *H *represents a homozygous site and *T *a heterozygous site. Recall that *T_i _*is the number of heterozygous sites in the genotype string, and those sites appear at indices *t_j _*where 1 ≤ *j *≤ *T_i_*. For this genotype there are  pairs of haplotypes that phase the given genotype. Represent each pair by setting *T_i _*- 1 binary variables

Note, that we are only interested in the origin of the children's haplotypes, rather than in the origin of *i*'s haplotypes, so the *p *and *m *can arbitrarily label either haplotype.

Since {*i*_1_, *i*_2_} between them have the parent genotype at every locus, one of them has origin *p *while the other has origin *m*, and similarly for {*i*_3_, *i*_4_}. For each locus, indicate the paternal origin of the allele for individuals *i*_1 _and *i*_3_, respectively with *Q_j _*and *S_j _*. Formally, *Q_j _*= 1 if both  and  while *Q_j _*= 0 otherwise. Similarly, *S_j _*= 1 if both  and  while *Q_j _*= 0 otherwise. Define *R_j _*as the minimum recombination count before locus *j*. Notice that  sets the origin of all the child haplotypes, therefore , since all preceding homozygous loci can have the same origin as locus *t*_1_.

From *t_j _*to *t*_*j*+1 _we have two cases:

1. If , then  and , by the alternating construction of children *i*_3 _and *i*_4 _as compared with *i*_1 _and *i*_2_.

2. Similarly, if , then  and .

Furthermore, regardless of the number of homozygous loci separating *t_j _*and *t*_*j*+1_, the number of recombinations can only be increased. Therefore, we have the recursion

proving the lemma.   □

After applying the mapping, the haplotype probability turns out to have a coefficient that is independent of the haplotype assignment to the non-founding parent of the proxy family. This coefficient can be computed in linear time from the genotype data using a Markov chain. The Markov chain has 16 states and has a transition step between each pair of neighboring loci. This small Markov model can be thought of in the sum-product algorithm as an elimination of the typed individuals in the proxy family; alternatively, it is also equivalent to peeling-off the typed proxy individuals in the Elston-Stewart algorithm [14]. Once we have this coefficient, independent of the haplotype assignment, it is clear that the likelihood and maximum probability haplotype problems also have haplotype solutions related proportionally to the genotype solution.

**Lemma 2**. *The mapping yields a haplotype problem instance having haplotype probabilities proportional to the haplotype probabilities of the genotype instance. Specifically, for all x*,

*where the proxy family transmission probability is a function of genotype g_i_, the recombination rate θ *≤ 0.5, *and of the transition matrices P *, *Q*_0110_, *and Q*_1001_,

*and O_j _indicates whether index j is odd, h_0 _is the number of homozygous loci that begin proxy parent's genotype, and h_j _is the number of consecutive homozygous loci after the j'th heterozygous locus where there are T_i _heterozygous loci for proxy parent i. The transition probabilities are given by P_ij _= θ*^*H*(*i, j*)^(1 *- θ*)^4*-H*(*i, j*) ^*where H*(*i, j*) *is the Hamming distance between inheritance states i and j. Let Q*_0110 _*be a transition matrix having non-zero recombination probabilities only in column *0110 *(i.e. Q*_0110, *i, j *_*= P_ij _when j = *0110*). Similarly, let Q*_1001 _*be a transition matrix with non-zero recombination probabilities only in column *1001.

*Proof*. Without loss of generality, assume that individuals *i *∈ *G *are all fathers in their proxy family. This is simply for convenience of notation.

Let *x *be any fixed assignment of haplotypes to all the individuals in the pedigree. When conditioning on the assigned haplotypes for individual *i*, the probability of the proxy family of *i *is independent of the probability for the rest of the pedigree. Since we can say this for all the proxy families, the terms in the probability for the pedigree individuals in set *I *(i.e. those also in the genotype pedigree) are equal to the probability on the genotype data in the genotype pedigree. Therefore, we write that

The sum over vector s can be split into sums over the component pieces. The sums involving the  can be distributed into the product over *k*, since that is the only place they are used. Let . We easily see that , since there are two ways to inherit the 1-allele from the mother, and all of them are compatible.

Let *p_t_*(*i*) be the transmission probability for the proxy family, defined as

View this probability as a Markov chain along the genome with a state space of size 2^4 ^where each state indicates the inheritance of (). The transition probabilities are given by *P_ij _*= *θ*^*H*(*i, j*)^(1 - *θ*)^4-*H*(*i, j*) ^where *H*(*i*, *j*) is the Hamming distance between inheritance states *i *and *j*. By design, the transitions allowed by the data have an unusual structure dictated by the heterozygous loci of the proxy parent. Specifically, at a heterozygous locus, there is exactly one inheritance state that satisfies the children's haplotypes. At homozygous loci, all the inheritance states are allowed. So, we compute this probability using the *l*-state transition probabilities to determine the contribution of a particular stretch of *l *homozygous loci that are followed by a heterozygous locus. Notice that the heterozygous locus has, as inheritance indicators, either (0, 1, 1, 0) or (1, 0, 0, 1), and these alternate between consecutive heterozygous loci.

Let *Q*_0110 _be a transition matrix having non-zero recombination probabilities only in column 0110 (i.e. *Q*_0110,*i, j *_= *P_ij _*when *j *= 0110). Similarly, let *Q*_1001 _be a transition matrix with non-zero recombination probabilities only in column 1001. Let *h*_0 _be the number of homozygous loci that begin proxy parent's genotype and let *h_j _*be the number of consecutive homozygous loci after the *j*'th heterozygous locus where 1 ≤ *j *≤ *T_i _*and *T_i _*is the number of heterozygous loci for proxy parent *i*. Now, we can write the transmission probability in terms of matrix operations

where *Z_j _*indicates whether the *j*'th heterozygous locus has inheritance indicators (0, 1, 1, 0). The column vector of ones at the end simply sums all final state probabilities to obtain the total probability.

Finally, notice that the two heterozygous inheritance states (0, 1, 1, 0) and (1, 0, 0, 1) are arbitrarily labeled. The main feature is that these states alternate at heterozygous loci, and it does not matter which one occurs first. So, we can write *p_t_*(*i*) as in the statement of the lemma in terms of *O_j _*which indicates the event that *j *is odd. Now we have a quantity that is a function of the genotype data and not dependent on the haplotypes under consideration.   □

**Corollary 3**. *The mapping yields a haplotype problem instance having a likelihood and maximum probability proportional, respectively, to the likelihood and maximum probability of the genotype instance. Specifically*,

and

*where p_t_(i) is proxy family i's transmission probability as defined in Lemma 2*.

*Proof*. Lemma 2 shows that *X *is independent of the coefficient of proportionality between the haplotype probability and the genotype probability. Therefore, this coefficient factors out of both the likelihood and the maximum probability equations.   □

Although this reduction establishes the hardness of these haplotype pedigree problems, it does so by constructing children whose haplotypes require many recombinations and would be extremely unlikely to occur naturally. Accordingly, we suspect that realistic instances of these haplotyping problems may provide more information about the locations of recombinations than genotype instances.

## Algorithms and Accuracy of Estimates

One indication that the haplotype problem might be practically more tractable is the amount of information in the haplotype data relative to the genotype data. To understand this, we can consider a pedigree with a fixed set of sampled individuals. Assume that there are two input data sets available, either the haplotype or the genotype data, for all the sampled individuals. Note that the alleles observed will be identical in both the haplotype and genotype data, so we are interested in the distribution that these data impose on the inheritance probabilities. By comparing the accuracy of the recombination estimates under these two data sets, we can get an idea for how useful the respective probability distributions are.

Let *R_j _*be a random variable representing the number of recombinations in the whole pedigree that occur between loci *j *- 1 and *j*. Similar to our notation before, . We want to compute the distribution of *R_j _*under both the genotype and haplotype inheritance probability distributions. These two inheritance distributions are different precisely because there are haplotypes and inheritance paths that are consistent with the genotype constraints but disallowed by the haplotype constraints.

These distributions are obtained by constructing a hidden Markov model for the linkage dependencies along the genome. At each locus, the HMM considers the constraints given by either the haplotype or genotype data (i.e. the haplotype data HMM is a variation on the Lander-Green algorithm [15]). We first use the forward-backward algorithm to compute the marginal inheritance probabilities for each locus using a hidden Markov model. Once we have the marginal probabilities, we can easily obtain the distribution for *R_j _*.

### General Haplotype and Genotype HMMs

The likelihood can be modeled using a hidden Markov model along the genome with inheritance paths as hidden states. An *inheritance path *is a graph with nodes being the alleles of individuals and directed edges between alleles that are inherited from parent to child. The transition probabilities are functions of *θ *and the number of recombinations between a given pair of inheritance graphs.

Given the data, we compute the marginal inheritance path probabilities at each site by using the forward-backward algorithm for HMMs. Sobel and Lange described a method for enumerating the inheritance paths compatible with the allele data observed at each locus [16]. There are at most *k *= 2^2|*I*\*F*| ^inheritance paths when *I*\*F *is the set of non-founder individuals, and both the forward and backward recursions do an *O*(*k*^2^) calculation at each site.

To compute the analogous probability for haplotype data, we use a similar HMM. For haplotypes, the hidden states must consider the haplotype orientations, which specify the parental origins of all the observed haplotypes. Notice that these orientations are not equivalent to inheritance paths, since they only specify inheritance edges between haplotyped individuals and their parents. For each of the 2^2|*H*| ^haplotype orientations, where *H *is the set of haplotyped individuals, we enumerate the inheritance paths compatible with the haplotype alleles, their orientations, and the pedigree relationships. Alternatively, each of the inheritance paths enumerated for the genotype algorithm induces a particular orientation on the haplotypes heterozygous for that locus (i.e. parental origin of the entire haplotype). Thus, the hidden states for the haplotype HMM are the cross-product of the orientations and the inheritance paths.

The haplotype HMM has transition probabilities that are nearly identical to the genotype HMM with the exception that transitions between inheritance paths with different haplotype orientations have probability zero. Recombinations are only allowed when they do not occur between typed haplotypes.

The forward-backward algorithm is also used on the haplotype HMM. However, there are 2^2(|*I*|+|*H*|-|*F*|) ^hidden states, yielding a slightly slower calculation. Fortunately, the haplotype recursions can be run simultaneous with the genotype recursions, meaning that the inheritance paths need only be enumerated once.

### Haplotype Likelihoods in Linear Time

There is one obvious instance of the haplotyping problems where there are polynomial-time algorithms. Even though it is impractical to assume that we can sample genetic material from deceased individuals in a multi-generational pedigree, for a moment, let us consider the case where all the individuals in the pedigree are haplotyped.

The Elston-Stewart algorithm [14] for genotype data has a direct analogue for haplotype data. This algorithm calculates the likelihood via the belief propagation algorithm by eliminating individuals recursively from the bottom up. Each individual is "peeled off", after their descendants have been peeled off, by using a forward-backward algorithm on the HMM for the mother-father-child trio.

The haplotype version of this algorithm is linear when all the individuals are haplotyped, since each elimination step is conditionally independent of all the others. Given the parents' haplotypes, regardless of which was inherited from which grand-parent, the probability of the child's haplotype is independent of all other trios. Therefore, we can take a product over the likelihoods for all the trios, and compute each trio likelihood using a 4-state HMM. Then for *k *non-founding individuals, and *l *loci, this algorithm has *O*(*kl*) running time.

This same intuition carries through to the minimum recombination problem, and each trio can be considered independent of the others. This contrasts with the genotype minimum recombination problem which is known to be hard, even when all the individuals are genotyped [7].

## Results

To simulate realistic pedigree data, SNPs were selected from HapMap that span 100 mb on both sides of a loosely-linked pair of sites. There are 40 SNPs total, with 20 tightly linked SNPs on each side of a strong recombination breakpoint having *θ *= 0.25. The haplotypes for these SNPs were selected randomly from HapMap. Pedigree haplotype and genotype data were simulated for each child by uniformly selecting one of the parental alleles for the first locus, and subsequent loci were selected on the same parental haplotype with probability *θ_j _*for each locus *j*. Inheritance was simulated for 500 simulation replicates.

The simulation yielded completely typed pedigrees. For each pedigree, we removed the genotype and haplotype information for increasing numbers of untyped individuals. For each instance of a specific number of untyped individuals, two values were computed on the estimated number of recombinations between the central pair of loci: the haplotype and genotype accuracies. Accuracy was computed as a function of the *l*_1 _distance between the deterministic number of recombinations and the calculated distribution. Specifically, accuracy was 2 - Σ_*i*≥0 _|*x*_*i *_- *a_i_*|, where *x_i _*was the estimated probability for *i *recombinations and *a_i _*was the deterministic indicator of whether there were *i *recombinations in the data simulated on the pedigree.

**Figure 3:**
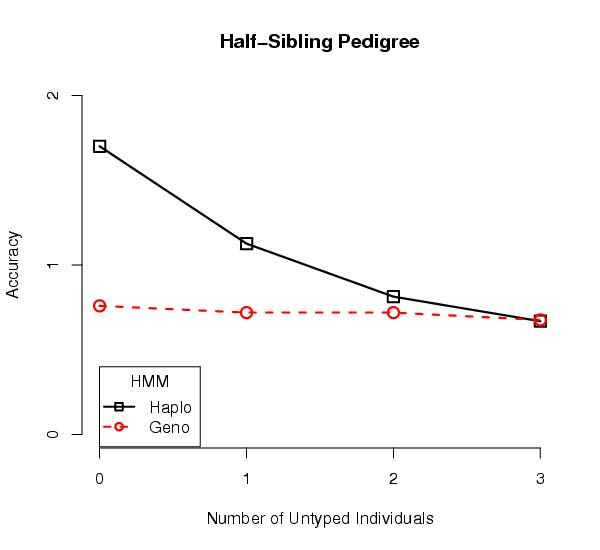
**Predicting Recombinations for Half-Siblings**. This is the average accuracy for predictions from a pedigree with two half-siblings and three parents. Five hundred simulation replicates were performed, and the average accuracy of estimates from the haplotype data is superior to those from genotype data. However, as the number of untyped founders increases, in both cases, the accuracy of estimates from haplotype data drop relative to the accuracy from genotype data. The accuracies of genotype and haplotype estimates appear to converge.

**Figure 4:**
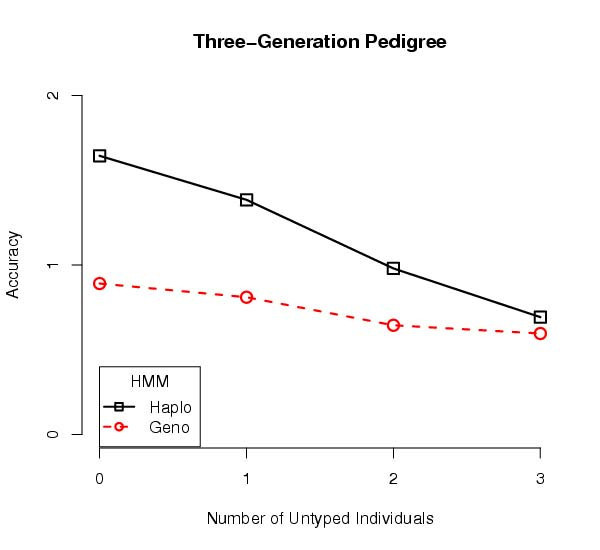
**Predicting Recombinations for Three Generations**. This figure shows accuracy results from a six-individual, three-generation pedigree. Again, five hundred simulation replicates were performed, and the average accuracy of estimates from the haplotype data is superior to those from genotype data. Once again, as the number of untyped founders increases, the accuracy of estimates from haplotype data drop relative to the accuracy from genotype data. The accuracies of genotype and haplotype estimates appear to converge.

In all the instances we observed a trend where the best accuracy was obtained with haplotype data where everyone in the pedigree was haplotyped. For example, a five-individual pedigree with two half-siblings is shown in Figure 3. With the three founders untyped, the haplotype data yielded similar accuracy as the genotype data. Consider a three-generation pedigree having two parents, their two children, an in-law, and a grandchild for a total of six individuals, three of them founders. This pedigree has a similar trend in accuracy as the number of untyped founders increases, Figure 4. As the number of untyped individuals increases, the accuracies of genotype and haplotype estimates appear to converge.

## Discussion

Sequencing technologies would seem to solve the phasing problem by yielding haplotype data. However, if we wish to consider diploid inheritance with recombination, the phasing problem remains, even when we are given chromosome-length haplotype data. This is demonstrated by reduction of the phasing problem for genotypes to the phased version of the same problem for three common pedigree problems. This theoretical result is due largely to the unavailability of genetic material for deceased individuals.

Three pedigree calculations were discussed: likelihood, maximum probability, and minimum recombination. Each of these calculations on haplotype data have the same computational complexity as the same computation on genotype data. In the worst case, it takes only a single generation to remove the correlation between sites in the haplotype. This worst case provided the reduction that proves the the complexity results for the haplotype computations, and it worked equally well for all three pedigree computations. The worst-case is not biologically realistic, since it requires roughly 2(*m *- 1) recombinations for *m *sites in 4 meioses. This is very unlikely to occur under typical models for inheritance. To investigate more likely scenarios, sequences were simulated in a region of the genome surrounding a recombination breakpoint. From haplotype and genotype data, we estimated the distribution of the number of recombinations at the breakpoint and compared the estimates to the ground-truth for accuracy.

When typing everyone in the pedigree, the estimates from haplotype data were very accurate, because the haplotype data provides enough constraints to determine where the recombinations must have occurred. With decreasing numbers of typed individuals, the accuracy of haplotype-based estimates dropped until it seemed to converge to the genotype accuracy due to a lack of constraints. From the structure of the calculations, we observed that with fewer typed individuals there were more haplotype orientations to consider, and the haplotype calculation more closely resembled the genotype calculation. However, the haplotype calculation had more constraints and lost accuracy at a slower rate.

Several interesting open problems remain. First, approximation algorithms might be a useful approach for haplotypes on pedigrees. The existence of a linear-time algorithm when all individuals are haplotyped may suggest that the general haplotype problem instance could be amenable to approximation algorithms. Second, these proofs apply when there is no missing data in a genotyped individual (i.e. a proxy parent).

The proof requires knowing whether the proxy parent is heterozygous or homozygous at each locus, and this is unknown when there is missing data. Third, there is an interesting case of mixed haplotypes and genotypes. For this case to be interesting, the ends of haplotypes must occur at different locations in different individuals in the pedigree. Otherwise, the haplotypes that start and end at the same positions in all individuals can easily be converted into multi-allelic genotypes, with an allele for each haplotype. The mixed haplotype-genotype problem is not amenable to the proof techniques used here. However, the haplotype HMM in Section can easily be revised to handle the mixed case. This is important because the data produced by single polymer sequencing is more likely to resemble the mixed case than either the haplotype or the genotype cases.

## Authors' contributions

BK concieved of the problem, proved the results, and implemented the algorithms.

## Competing interests

The authors declare that they have no competing interests.
